# Subarachnoid Contrast Hyperdensity Following Pelvic Trauma Mimicking Diffuse Subarachnoid Hemorrhage

**DOI:** 10.7759/cureus.10460

**Published:** 2020-09-15

**Authors:** Saptarshi Biswas, Arpit Amin

**Affiliations:** 1 Surgery, Grand Strand Medical Center, Myrtle Beach, USA

**Keywords:** contrast hyperdensity, dural tear, pelvic trauma, subarachnoid hemorrhage

## Abstract

We present a case of a 54-year-old male who was involved in a motorcycle accident. His head computed tomography (CT) scan on arrival at our Level 1 institution was positive for hyperdensity suspicious for subarachnoid hemorrhage (SAH). Spine CT showed anterior compression fractures of T7-T9 vertebral bodies along with the presence of contrast within the subarachnoid space in the thoracic and lumbar spine, raising suspicion for a dural tear. CT of the chest, abdomen, and pelvis revealed open book pelvic fracture, left sacral ala fracture extending into the left sacroiliac joint and S1 neural foramen, coccygeal fracture, and extraperitoneal bladder rupture.

This rare case report highlights the possibility of a spinal meningeal tear in severe pelvic trauma with concomitant bladder injury as a pathway of contrast entry into the normally impermeable cerebrospinal fluid (CSF) space mimicking traumatic subarachnoid hemorrhage.

## Introduction

There are isolated reported cases in the literature describing radiographic mimics of subarachnoid hemorrhage (SAH). The etiology has been attributed to several causes, including pyogenic leptomeningitis, idiopathic intracranial hypertension, pseudotumor cerebri, diffuse cerebral edema, septic shock, metabolic derangement, computed tomography (CT) appearance of intrathecally administered contrast material, and leakage of high-dose intravenous contrast medium into the subarachnoid space [1-5]. Angiography causing brain parenchymal and intraventricular contrast enhancement being mistaken for intraparenchymal hemorrhage has also been reported [6]. We report a unique case in which concomitant bladder injury and sacral dural tear led to contrast enhancement of the brain parenchyma, mimicking subarachnoid hemorrhage.

## Case presentation

A 54-year-old male was transferred to our institution as a Level 1 trauma from an outside hospital. He was riding his motorcycle and was struck by a truck at high speed, leading to a collision with a guardrail. He was wearing a helmet, denied any loss of consciousness, and did not have any amnesia. Upon arrival, he complained of pain in the lower part of his abdomen, lower back, and bilateral lower extremities.

The patient’s medical and surgical history was significant for hypertension, dyslipidemia, Lyme disease, carpal tunnel syndrome, and left elbow and right knee surgery. The patient denied any allergies. The patient did not take any home medications and denied taking any anticoagulant or antiplatelet agent. The patient’s social history was not significant for any recreational drugs.

The patient’s vital signs on arrival were: temperature (T) 96.2, heart rate (HR) 108, blood pressure (BP) 110/77, respiratory rate (RR) 24, and oxygen saturation (O2 sat) 100%. The primary survey revealed an intact airway, clear breath sounds bilaterally, palpable central and distal pulses, no obvious deformities, a Glasgow Coma Scale (GCS) of 15, and equal, reactive, non-dilated pupils bilaterally. The secondary survey was significant for diffuse abdominal tenderness, pelvic instability, suprapubic tenderness, gross blood at the urinary meatus, scrotal hematoma, perirectal hematoma, normal rectal tone, lumbar spine tenderness, and abrasions on his upper and lower extremities.

Labs on presentation were as shown in Table 1. 

**Table 1:** Labs on presentation WBC: white blood cell; Hgb: hemoglobin; Hct: hematocrit; Plt: platelet count test; BUN: blood urea nitrogen; BG: blood glucose; T.bil: total bilirubin; AST: aspartate aminotransferase; ALT: alanine transaminase; ALP: alkaline phosphatase; PT: prothrombin time; INR: international normalized ratio; PTT: partial thromboplastin time

WBC	17
Hgb	11.6
Hct	34.4
Plt	142
Na	139
K	4.1
Cl	110
HCO3	15
BUN	15
Cr	0.99
BG	175
Ca	7.4
T.bil	0.4
AST	32
ALT	21
ALP	37
PT	11.5
INR	1.05
PTT	29.8

The chest X-ray was normal. The pelvis X-ray showed a tile-type C1 open book pelvic fracture. A pelvic binder was placed. The focused assessment with sonography in trauma (FAST) exam was positive for bladder injury.

Bedside urethrocystogram showed extraperitoneal bladder rupture. Head CT showed hyperdensity in the subarachnoid space, raising suspicion for traumatic subarachnoid hemorrhage. Spine CT showed anterior compression fractures of the T7-T9 vertebral bodies along with the presence of contrast within the subarachnoid space in the thoracic and lumbar spine, raising suspicion for a dural tear. CT of the chest, abdomen, and pelvis revealed open book pelvic fracture, left sacral ala fracture extending into the left sacroiliac joint and S1 neural foramen, coccygeal fracture, and extraperitoneal bladder rupture.

The patient was taken to the operating room. A flexible cystourethroscopy performed revealed no injury in the urethra but did show a rent approximately 6 cm in dimension in the left anterolateral portion of the bladder. Reapproximation of the mucosa and submucosal layers of the bladder was done with a running 2-0 chromic suture. The detrusor was then approximated using 2-0 Vicryl interrupted figure-of-eight sutures. Methylene blue test following the repair revealed no leaks. A Foley catheter was left in place. Anterior plating of the pubic symphysis was performed and the anterior pelvis was placed in an external fixator. The patient was started on empiric antibiotics.

A repeat non-contrast head CT on hospital Day 2 revealed persistent contrast within the subarachnoid space. Magnetic resonance imaging (MRI) of the thoracic spine showed no acute fractures. On hospital Day 3, the patient underwent open reduction and internal fixation of the posterior pelvic ring and the left sacral ala fracture along with percutaneous pinning of the left sacroiliac joint.

The patient’s hospital course was complicated by delirium on hospital Day 4, which improved after stopping narcotic-based patient-controlled analgesia (PCA). The Foley catheter was kept in place for two weeks postoperatively and a repeat cystogram on hospital Day 10 showed patent bladder repair. The patient was kept non-weight-bearing on the left lower extremity for eight weeks. The patient was discharged home on hospital Day 13 with home physical therapy. The patient was treated with empiric antibiotics for two weeks postoperatively. Postoperative follow-up has not revealed any signs of meningitis.

The following is a pictorial description eliciting the spread of intravenous contrast material through the meningeal tear at the level of sacrum following significant pelvic injury and subsequent presentation as subarachnoid hyperdensity.

**Figure 1:**
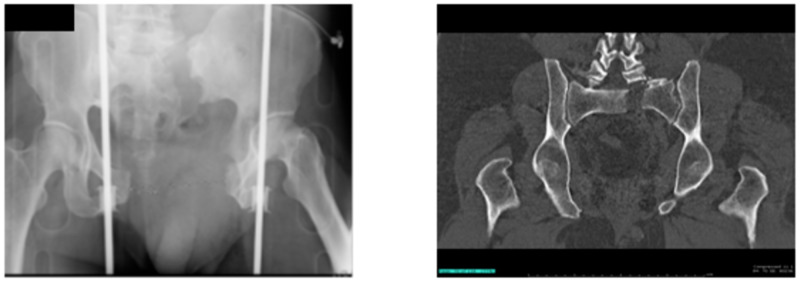
Tile Type C1 pelvic fracture (left); posterior pelvic disruption (right)

The pelvic X-ray on the day of admission showed an open-book fracture involving the pelvis with significant diastasis of the pubic symphysis (Figure 1). The CT scan of the pelvis showed significant comminution of the left sacral wing extending into the left sacroiliac joint and left S1 neural foramen (Figure 1).

**Figure 2:**
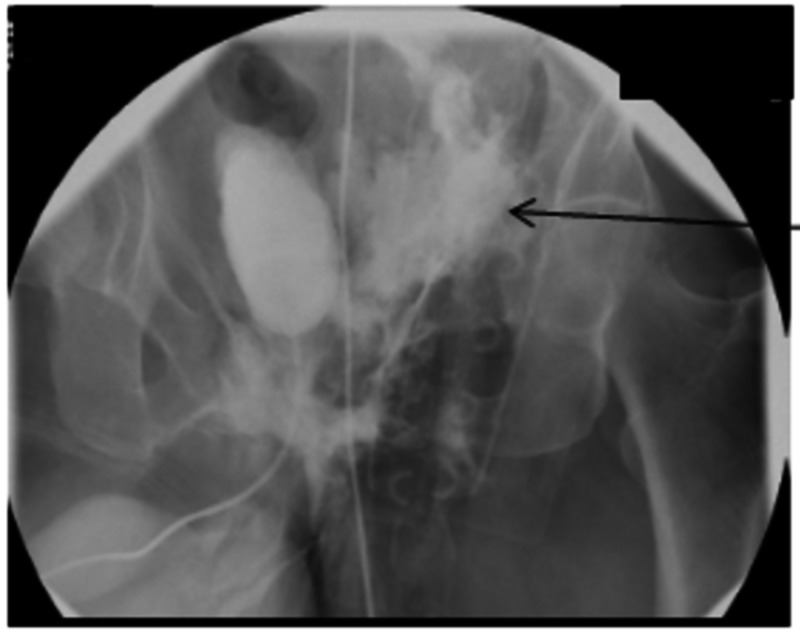
Extravasation on the left side of the bladder neck (arrow)

Urethrocystogram showed an extraperitoneal bladder rupture at the left side of the bladder neck (Figure 2). 

**Figure 3:**
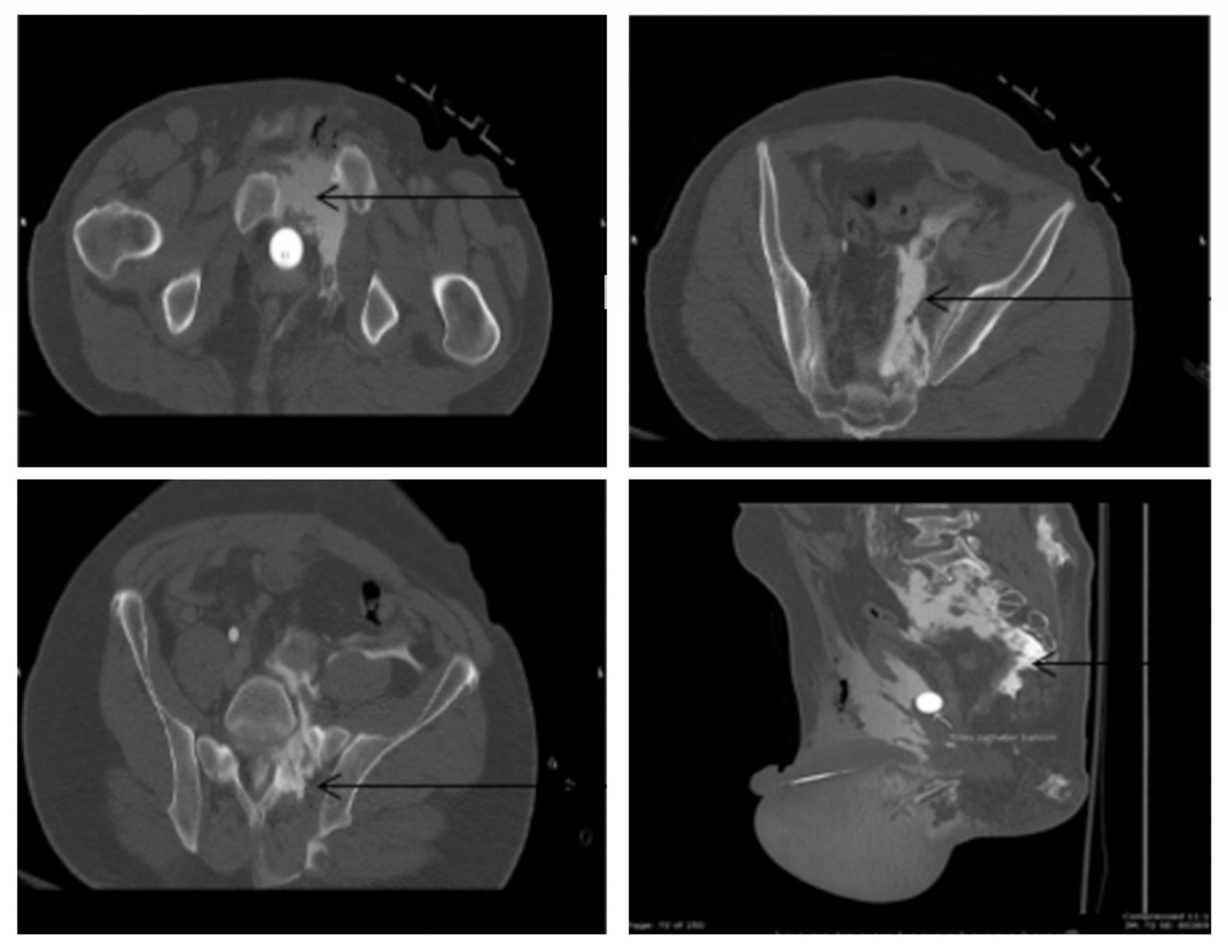
Contrast in the extra-peritoneal space (arrow, top left); contrast tracking to the space around the site of the bladder rupture in the retroperitoneum at the level of the sacrum (arrow, top right); contrast entering the lumbar subarachnoid space (arrow, bottom left); contrast ascending along the subarachnoid space due to the dural tear (arrow, bottom right)

The pathway of the contrast from the extraperitoneal bladder rupture to the level of the meningeal tear associated with the sacral fracture is shown in Figure 3.

**Figure 4:**
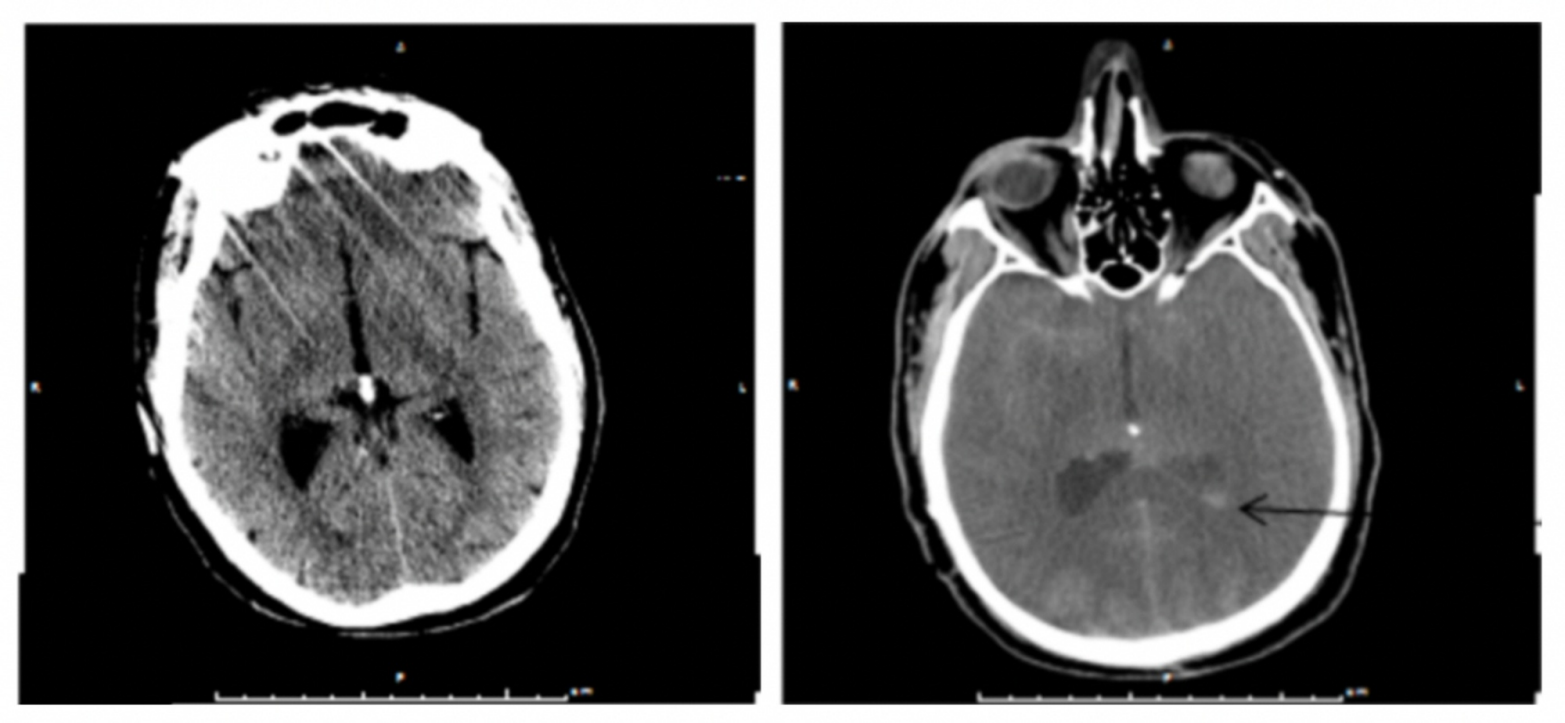
Initial head CT on presentation showing no subarachnoid hyperdensity (left); six-hour interval head CT showing intraventricular hyperdensity (arrow, right) CT: computed tomography

Extensive subarachnoid hyperdensity was found on a CT scan of the head, raising concern for traumatic subarachnoid hemorrhage versus the possibility of subarachnoid contrast material (Figure 4). 

**Figure 5:**
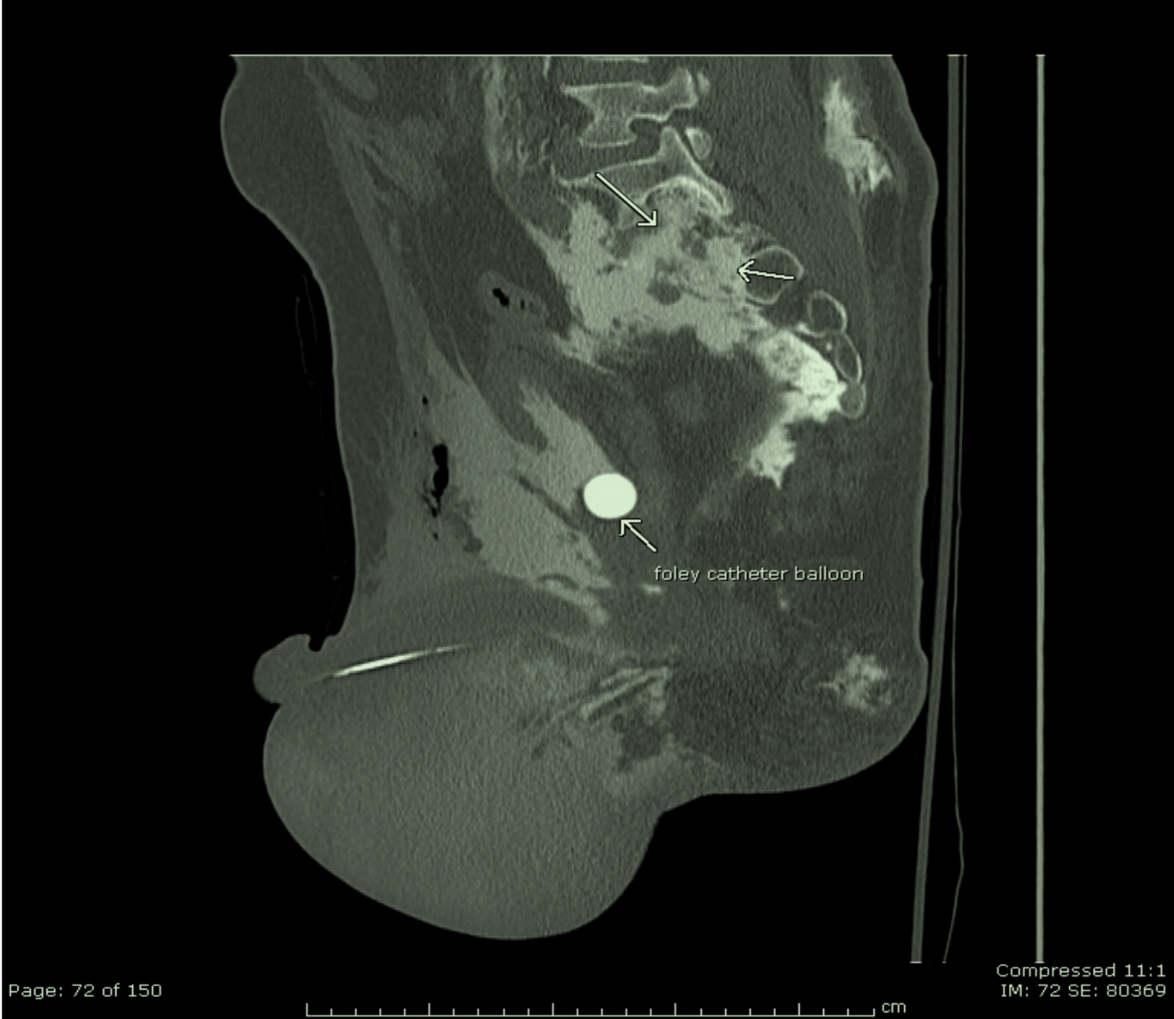
CT of the thoracolumbar spine with coronal and sagittal reconstructions CT: computed tomography

Extensive contrast material was seen in the subarachnoid space following the anterior compression fractures of T7-T9 (Figure 5).

## Discussion

It is known that approximately 16% of patients with pelvis fractures have associated genitourinary injuries [7]. Therefore, one should maintain a high index of suspicion for genitourinary injuries in patients with pelvic fractures. Bladder injuries are associated with macroscopic or microscopic hematuria. In the setting of pelvic fractures, extraperitoneal bladder rupture is the most common type of bladder injury. Bladder injuries can be confirmed via a conventional cystogram or CT cystogram. Operative intervention is indicated in an extraperitoneal bladder injury if the injury is extensive or if the associated pelvic fractures require fixation with hardware [8].

When there is an injury to the posterior component of the pelvic ring, the possibility of a nerve injury should be taken into consideration [1]. In this case, a posterior pelvic fracture resulted in a meningeal tear at the level of the second sacral ala. It is known that the spinal dura mater forms the tube downwards through the vertebral canal between the foramen magnum and the sacrococcygeal complex. Inside the vertebral canal, the dural attachments to the bone are to the posterior bodies of the second and third cervical vertebra and to the posterior body of the second sacral segment [9]. A severe injury of the sacrum can lead to disruption of the underlying meninges.

There are reported cases in the literature showing intraventricular enhancement after intra-arterial injection of iodinated contrast or gadolinium contrast [6,10]. The blood-brain barrier is an anatomic and physiologic barrier separating the brain parenchyma and he intravascular space [6]. The tight junction between the epithelial cells within the brain parenchyma is relatively impermeable [11]. The impermeability can be disrupted under pathologic processes like a tumor, ischemia, an overdose of contrast material, or intra-arterial injection of contrast material [6,10-11].

Some authors have used the term pseudo SAH for these CT mimics of SAH [1,5,12]. Given et al. [2] report the CT findings of seven patients with diffuse cerebral edema who presented with increased attenuation in the basilar cisterns resembling SAH. True SAH were excluded on the basis of autopsy and lumbar puncture findings. Avrahami et al. noted pseudo-SAH in a case series of nontraumatic comatose patients [12]. Osborn et al. [5] and Spiegel et al. [4] have mentioned increased attenuation of the falx and tentorium in association with cerebral edema. Oh et al. [13] describe a case of contrast enhancement of the subarachnoid space simulating SAH following lumbar percutaneous epidural neuroplasty (L-PEN). Sarohia et al. [14] describe a case where the migration and distribution of silicone along the intracranial visual pathway and, eventually, the whole of the ventricular system resulted from an intraocular silicone injection for complex retinal detachment.

In our patient, subarachnoid hyperdensity raised the concern for traumatic brain injury. However, a review of the initial CT scan of the head performed six hours earlier at the outside hospital did not reveal any intracranial hemorrhage. The normally impermeable dura mater and arachnoid mater were disrupted at the level of the sacral fracture. In the presence of a concomitant extraperitoneal bladder rupture, this disruption of the meninges led to the entry of the contrast into the subarachnoid space. This led to a paradoxical finding of subarachnoid hyperdensity within the brain, in our case mimicking a traumatic subarachnoid hemorrhage. The finding was independently confirmed by the neurosurgery and the neuroradiology team.

## Conclusions

This case report highlights the possibility of a spinal meningeal tear in severe pelvic trauma with a concomitant bladder injury as a pathway of contrast entry into the normally impermeable cerebrospinal fluid (CSF) space, mimicking a traumatic subarachnoid hemorrhage.
